# Neurobiological response to EMDR therapy in clients with different psychological traumas

**DOI:** 10.3389/fpsyg.2015.01614

**Published:** 2015-10-27

**Authors:** Marco Pagani, Giorgio Di Lorenzo, Leonardo Monaco, Andrea Daverio, Ioannis Giannoudas, Patrizia La Porta, Anna R. Verardo, Cinzia Niolu, Isabel Fernandez, Alberto Siracusano

**Affiliations:** ^1^Institute of Cognitive Sciences and Technologies, Consiglio Nazionale delle RicercheRome, Italy; ^2^Laboratory of Psychophysiology, Chair of Psychiatry, Department of Systems Medicine, University of Rome “Tor Vergata”Rome, Italy; ^3^Chair of Psychiatry, Department of Systems Medicine, University of Rome “Tor Vergata”Rome, Italy; ^4^Psychiatry and Clinical Psychology Unit, Department of Neurosciences, Fondazione Policlinico “Tor Vergata”Rome, Italy; ^5^EMDR Italy AssociationBovisio Masciago, Italy

**Keywords:** psychotherapy, EMDR, EEG, bilateral ocular stimulation, chronic psychological trauma, prefrontal cortex activation

## Abstract

We assessed cortical activation differences in real-time upon exposure to traumatic memory between two distinct groups of psychologically traumatized clients also in comparison with healthy controls. We used electroencephalography (EEG) to compare neuronal activation throughout the bilateral stimulation phase of Eye Movement Desensitization and Reprocessing (EMDR) sessions. We compared activation between the first (T0) and the last (T1) session, the latter performed after processing the index trauma. The group including all clients showed significantly higher cortical activity in orbito-frontal cortex at T0 shifting at T1 toward posterior associative regions. However, the subgroup of clients with chronic exposure to the traumatic event showed a cortical firing at both stages which was closer to that of controls. For the first time EEG monitoring enabled to disclose neurobiological differences between groups of clients with different trauma histories during the reliving of the traumatic event. Cortical activations in clients chronically exposed to traumatic memories were moderate, suggesting an association between social and environmental contexts with the neurobiological response to trauma exposure and psychotherapy.

## Introduction

Non-invasive brain activity recording has been used to investigate anatomical and functional changes occurring in post-traumatic stress disorder (PTSD). Functional studies have described significant neurobiological alterations in patients with PTSD while both reliving psychological traumas (Shin et al., 1999, 2004; Lanius et al., 2002, 2003; Lindauer et al., 2004; Britton et al., 2005; Osuch et al., 2008) and during resting state (Todder et al., 2012; Lee et al., 2014). The brain areas more frequently implicated in PTSD studies (hippocampus, amygdala, medial prefrontal cortex, anterior and posterior cingulate, and temporal cortex; Francati et al., 2007) belong to the limbic system, known to be involved in processing both positive and negative emotions. Clients exposed to psychologically traumatic events lack the capability to process traumatic memories, with representing a reduced medial frontal cortex and anterior cingulate inhibition over a hyperaroused amygdala the core functional mechanism of symptoms.

The neurobiological correlates of psychotherapies targeting PTSD assessed both during the symptomatic phase of the disease and after therapeutic interventions have been recently investigated (Lansing et al., 2005; Lindauer et al., 2005; Felmingham et al., 2007; Pagani et al., 2007; Peres et al., 2007; Bryant et al., 2008; Lindauer et al., 2008; Rabe et al., 2008; Roy et al., 2010; Peres et al., 2011; Kluetsch et al., 2014). These studies identified brain cortical and sub-cortical changes associated to therapy providing information about their impact on regions processing emotions. However, little is known about the neurobiological effect of psychotherapies in clients suffering different traumas and with different levels of exposure to traumatic memory over time. Furthermore, in previous studies the reported information were limited to static conditions, with functional and anatomical changes recorded before and after treatment, without any real-time description of the dynamics of neuronal activity during psychotherapy sessions. Recently, an EEG investigation (Pagani et al., 2012) has broken fresh ground disclosing the brain cortical activity modifications occurring during sessions of Eye Movement Desensitization and Reprocessing (EMDR), one of the evidence-based treatments for PTSD, included in many international guidelines (United Kingdom Department of Health, 2001; Dutch National Steering Committee Guidelines Mental Health Care, 2003; American Psychiatric Association, 2004; INSERM, 2004). Moreover EMDR has been proven to be more successful than pharmacotherapy in achieving sustained symptoms reductions (van der Kolk et al., 2007).

The aim of the present study was two-fold: (1) to use EEG to compare cortical activation changes occurring during bilateral stimulation phase of EMDR sessions between cohorts of clients and healthy controls (2) to investigate in two subgroups of clients exposed to different traumas and in different circumstances the possible specific neurobiological correlates of both initial psychological condition and psychotherapy effect.

## Methods and materials

### Subjects

Forty psychologically traumatized clients (CLI) were included in the study (Table 1). They were divided in two groups of 20 individuals each, based on type of traumatic event criteria.

**Table 1:** **Socio-demographic and clinical descriptive statistics**.

	**CTR (*n* = 20)**	**RM (*n* = 20)**	**SGP (*n* = 20)**	**Statistics**
**Gender**				
Women	11	14	11	$\chi_{2}^{2}=1.276$
Men	9	6	9	*p* = 0.535
Age	35.87 (10.32)	36.53 (12.11)	36.65 (14.49)	*F*_(2, 57)_ = 0.028
				*p* = 0.973
**Trauma**^a^				
Autodirect	11	15	7	$\chi_{2}^{2}=6.465$
Eterodirect	9	5	13	*p* = 0.036
Age at the trauma time	25.90 (12.89)	18.20 (14.77)	27.40 (14.61)	*F*_(2, 57)_ = 2.446
				*p* = 0.096
Time from trauma^b^	10.50 (8.04)	18.60 (15.01)	9.5	*T*_(38)_ = 2.127
				p_2−*tailed*_ = 0.042
**PTSD (current)**				
No	–	3^c^	7^c^	$\chi_{1}^{2}=2.133$
Yes	–	17	13	*p* = 0.144
**Comorbidity (Axis I)**				
No	–	8	12	$\chi_{1}^{2}=1.600$
Yes	–	12	8	*p* = 0.206
**Psychotropic drug**				
No	–	12	16	$\chi_{1}^{2}=1.905$
Yes	–	8	4	*p* = 0.168

Data are frequencies and mean (SD). Univariate results (Chi-square tests and ANOVAs) are also reported.

a RM vs. SGP: $\chi_{\text{1}}^{\text{2}}$ = 6.465, p = 0.011; RM vs. CTR: $\chi_{\text{1}}^{\text{2}}$ = 1.758, p = 0.185; SGP vs. CTR: $\chi_{\text{1}}^{\text{2}}$ = 1.616, p = 0.204.

b Due to the absence of the variability of SGP group, a t-test for independent sample was conducted to compare RM vs. CTR

c All these 10 patients had a current sub-syndromal PTSD (intrusive experiences of trauma during the last month without reaching a Full MINI-Plus PTSD diagnosis).

The first group (investigated in Rome: RM) included 20 clients visited by EMDR therapists in private practice (*n* = 10) and in the outpatients clinic of Tor Vergata University Hospital (*n* = 10) specialized in EMDR treatment following psychological traumatic events. The presence of major psychological trauma was assessed by experienced psychiatrists as independent assessors and included sexual abuse (*n* = 11), grief and loss trauma (*n* = 5), abortion related trauma (*n* = 2) and severe physical abuse (*n* = 2).

The second group (receiving EMDR by experienced therapists in San Giuliano di Puglia: SGP) was composed by 20 clients recruited among the population that suffered in year 2002 a devastating earthquake which caused the collapse of a primary school and killing 27 children and one schoolteacher. The clients of this group belonged to the same social and territorial context, seven were survivors of the crash, five were their psychologically traumatized relatives and eight were parents, brothers and sisters of children dead in the collapse of the school roof. All subjects had a concurrent traumatic memory related to the natural disaster by itself. None of the subjects underwent previous trauma-focused psychotherapy and 30 out of 40(75%) of them had current clinically evident PTSD diagnosis (Table 1).

Twenty age- and sex-matched healthy subjects free of any mental disorder (Table 1) and aware of the study agreed of their own free wills to participate as controls. They underwent the same EMDR protocol and psychological assessments, with the exclusion of CAPS. In the controls the index trauma was represented by the traumatic memory with highest impact (road trauma, *n* = 9; grief and loss trauma, *n* = 8; emotional trauma, *n* = 2; physical abuse, *n* = 1) and EMDR sessions focused on this life event.

The key distinctions between groups were: (i) the lack of trauma-related symptoms in the controls; (ii) common experience of the natural disaster and the temporal and environmental homogeneity of the SGP as compared to the more heterogeneous RM one.

Motivations for recruiting control subjects were the following: (i) to increase the robustness of the results adding a between-subjects analysis to the comparison of patients at T0 (first EMDR session) and T1 (last EMDR session); (ii) to cross-check that the cortical activation upon EMDR was not due to bilateral ocular stimulation by itself; (iii) to cross-check that the cortical activation upon EMDR was not merely due to exposure to traumatic memory, irrespectively of the pathological state.

The institutional ethics committee approved the study. Before implementing the study protocol all participants were given a description of the procedures and were asked to sign a written informed consent to participate in the study in accordance with the Declaration of Helsinki.

### Study design

The study was performed in quiet rooms, light and airy and a trained psychiatrist, not involved in EMDR therapy, performed clinical evaluation. During the assessment session clinicians evaluated the impact of the major psychological trauma and persistence over time of the related symptoms. When possible, subjects were interviewed with the Mini International Neuropsychiatric Interview-Plus (MINI-Plus; Sheehan et al., 1998) and Clinician-Administered PTSD Scale (CAPS; Weathers et al., 2001) whose completion required about 45 min. Due to the clients' refusal to receive long interviews it was not possible to perform CAPS and MINI-Plus in 10 clients of RM. All subjects were given self-administered psychological questionnaires [the Impact of Event Scale (IES; Horowitz et al., 1979), the Beck Depression Inventory (BDI; Beck and Steer, 1993), and the Symptom CheckList-90-Revised (SCL-90-R; Derogatis and Lazarus, 1994)] and then they entered the therapy room where EEG cap was positioned and EEG recording was continuously performed during EMDR therapy (T0). The following sessions were based on the same EMDR protocol and were performed until the last session (T1) in which the client reported no disturbance with Subjective Unit of Distress (SUD) = 0, Validity of Cognition (VOC) = 7 and clear Body Scan. At T1 clients underwent a second neuropsychological assessment with the exception of MINI-Plus. The therapeutic process required two to six once-weekly sessions to be successfully completed.

As for control subjects, the EEG recording of the whole EMDR session was performed only once right after the neuropsychological assessment (T0).

### EMDR procedure

The eight phases of the treatment have been described in details elsewhere (Pagani et al., 2012). In brief, an EMDR session begins with the identification of the worst image of the traumatic memory, the negative belief, the disturbing emotion and the body location of the disturbance. Then, the client is asked to focus on these components of memory and follow the fingers of the clinician performing for about 30 s a bilateral stimulation (BS) guiding her eyes from right to left with sets of 30 s. After each set the client shares what she/he has been noticing until the feedback reveals that memory is desensitized and reprocessed in a constructive and adaptive way. When the process is completed, the client reports to be able to think of the traumatic experience with no disturbing emotions, with a positive self-belief and with the body tension free. EMDR includes working with the trauma memory and with anxiety provoking present situations linked with the traumatic experience. Treatment is concluded when the client visualize her/himself in a situation in the future where she/he faces the same triggers, feeling no emotional discomfort and a completely true positive self-statement.

The goal of EMDR is to address past, present and future issues related to traumatic events and reprocess them. Once these issues are desensitized and reprocessed, post-traumatic symptoms show significant remission.

### Clinical assessment

#### Interviews

MINI-Plus, according to the DSM-IV criteria, assesses the presence of a wide range of psychiatric disorders including PTSD diagnosis.

CAPS measures frequency and intensity of PTSD symptoms rated for the last-week period. Seventeen items describe the classical PTSD cluster symptoms: re-experiencing, avoidance and numbing, and hyperarousal as well as symptoms associated with PTSD features. The CAPS total score ranging from 0 to 136 classifies PTSD as: 0–19, asymptomatic/few symptoms; 20–39, mild PTSD/subthreshold; 40–59, moderate PTSD/threshold; 60–79, severe PTSD symptoms; and ≥80, extreme PTSD symptoms.

#### Self-administered questionnaires

IES regards the response to stressful events during the past week tackling specifically areas of intrusion and avoidance. Total scores range from 0 to 75. Scores above 26 are considered to be clinically significant.

BDI measures symptoms of depression related to cognition and affection as well as to somatic changes bothering clients in the previous week (0 = not at all to 3 = severe). Total scores range from 0 to 63, with scores above 18 indicating moderate to severe depressive symptoms.

SCL-90 R reports symptoms of psychological problems in the last 7 days allowing to assess their frequency. Subjects rate the items using a five-point scale (1 = no problem to 5 = very serious). It has three global indexes measuring the extent or depth of individual's psychiatric disturbance; the total number of questions rated above one point and the intensity of symptoms.

A General Linear Model approach was performed to compare scores of CAPS, IES, BDI and SCL-90-R between groups (between subject-group: “CTR vs. RM vs. SGP”), between clients pre- and post-EMDR treatment (within subject-group: EMDR Treatment, ET) and to test the interaction effect between clients' group (“RM vs. SGP”: Group, G) and EMDR treatment (EMDR Treatment × Group, ET × G). *Post-hoc* tests were performed with Bonferroni confidence interval adjustment for multiple comparisons to define which variables contributed to the major effects. Statistical significance was set at *p* < 0.05.

### EEG procedure

The detailed EEG methodology and statistics has been described elsewhere (Pagani et al., 2011, 2012). In brief, 37-channel EEG was recorded throughout the whole 1-h EMDR sessions using a pre-cabled electrode cap. Data were exported to EDF using NPX Lab 2010 (www.brainterface.com). We selected and exported only the epochs in which BS (typically between 20 and 25 in each session and lasting about 30 s) were performed. A file lasting several min composed by concatenated BS periods was then created and analyzed. These merged epochs represented the period during EMDR sessions in which clients were both focussed on the worst image of the index trauma and performing rhythmic bilateral ocular movements. Data were analyzed in the EEGLAB environment (http://www.sccn.ucsd.edu/eeglab/index.html; Delorme and Makeig, 2004), digitally band-pass filtered between 1 and 45 Hz and re-referenced to average reference. Artifactual non-cerebral source activities (eye blinks and movements, microsaccadic, cardiac and muscle/electromyographic activity) were identified and rejected using a semiautomatic procedure based on Independent Component Analysis (Porcaro et al., 2009). To compute intracerebral electrical sources we used exact low resolution brain electromagnetic tomography (eLORETA) software (http://www.uzh.ch/keyinst/loreta.htm; Pascual-Marqui, 2007; Pascual-Marqui et al., 2011). Computations were made using the Montreal Neurological Institute (MNI; Montreal, Quebec, Canada) MNI152 template (Mazziotta et al., 2001), with the three-dimensional solution space restricted to cortical gray matter and hippocampi, as determined by probabilistic Talairach atlas (Lancaster et al., 2000). Intracerebral volume (eLORETA inverse solution space) was partitioned in 6239 cubic voxels of 5 mm in which electric activity is represented for each voxel. Anatomical labels as Brodmann areas (BAs) are also reported using MNI space, with correction to Talairach space (Brett et al., 2002). Images corresponded to the estimated neuronal generators of brain activity within each band (Frei et al., 2001). The ranges of frequency bands were: delta (δ), 1.5–4 Hz; theta (θ) 4–8 Hz; alpha (α) 8–12 Hz; beta 1 (β1) 12–20 Hz; beta 2 (β2) 20–30 Hz; gamma (γ) 30–45 Hz.

sLORETA/eLORETA package was used for electrical source imaging statistical analyses estimating the probability distribution for max-statistic and correcting for multiple testing (Nichols and Holmes, 2002). Exceedence proportion tests evaluated the significance of activity based on its spatial extent, obtaining clusters of supra-threshold voxels. Between-group comparisons of eLORETA current density distribution were performed using voxel-by-voxel log of *F* ratio test. Results corresponded, for each band, to maps of log-*F*-ratio statistics for each voxel, for corrected *p* < 0.05. Significant activations with a *p* < 0.01 at exceedence proportion tests, *F*-value over z-score and clusters containing more than 50 voxels for a single region of interest (ROI, see **Table 4**) were accepted.

## Results

Socio-demographic and clinical data are shown in Table 1. Ten clients did not fulfill the clinical criteria of current PTSD at MINI-Plus.

All 12 patients that used psychotropic drugs took SSRI (or serotoninergic drugs). Moreover: in one case (RM), lithium; in two cases (one RM and one SGP), valproate; in three cases (two RM and one SGP), low doses of atypical antipsychotics (two quetiapine, one RM and one SGP, and one olanzapine in RM, with a nighttime administration) as add-on antidepressant strategies of SSRIs/serotoninergic drugs; in six cases low doses of short-duration benzodiazepines (four RM and two SGP) as hypnotic.

Twenty patients had Axis I comorbidity. Among the 12 RM patients, seven had a major depressive disorder, one a bipolar I disorder, one a bipolar II disorder, five a panic disorder and two a generalized anxiety disorder. Among the eight SGP patients, six had a major depressive disorder, one a bipolar II disorder, four a panic disorder and one a generalized anxiety disorder. None of the clients showed any sign of dissociation.

All subjects were right-handed, except two controls, three RM clients and three SGP clients ($\chi_{2}^{2}=0.289$, *p* = 0.866).

### Psychopathological evaluation

At group level scores of CAPS, IES, BDI, and SCL-90-R, as well as their subscales, differed significantly at T0 between controls and clients (Table 2) rating PTSD symptoms from moderate to severe. Though RM had a trend of constantly higher scores only BDI-TOT and four subscales of CAPS, IES, BDI, and SCL-90-R differed significantly from SGP (Table 2).

**Table 2:** **Descriptive and univariate (pre-EMDR) statistics of psychopathological scales are showed**.

	**CTR (*n* = 20)**	**RM–pre (*n* = 20)**	**SGP–pre (*n* = 20)**	**ANOVAs**			
				**Main effect**	**Bonferroni *post-hoc***		
					**RM vs. CTR**	**SGP vs. CTR**	**RM vs. SGP**
CAPS–TOT	2.80 (6.41)	77.80 (17.77)	62.60 (23.99)	*F*_(2, 47)_ = 83.995; *p* < 0.0001	*p* < 0.0001	*p* < 0.0001	*p* = 0.092
CAPS–RE-EXP	1.00 (2.85)	17.90 (5.53)	13.25 (9.34)	*F*_(2, 47)_ = 27.360; *p* < 0.0001	*p* < 0.0001	*p* < 0.0001	*p* = 0.234
CAPS–AVO-NUM	0.65 (1.35)	25.50 (7.32)	17.90 (8.49)	*F*_(2, 47)_ = 63.357; *p* < 0.0001	*p* < 0.0001	*p* < 0.0001	*p* < 0.009
CAPS–H-AROU	0.80 (1.74)	15.30 (4.50)	14.35 (5.63)	*F*_(2, 47)_ = 64.579; *p* < 0.0001	*p* < 0.0001	*p* < 0.0001	*p* = 1.000
CAPS–ASSOC	0.00 (0.00)	7.30 (4.03)	8.00 (5.77)	*F*_(2, 47)_ = 21.960; *p* < 0.0001	*p* < 0.0001	*p* < 0.0001	*p* = 1.000
IES–TOT	4.25 (7.41)	41.65 (18.29)	30.70 (18.39)	*F*_(2, 57)_ = 30.483; *p* < 0.0001	*p* < 0.0001	*p* < 0.0001	*p* = 0.091
IES–INT	2.95 (6.44)	20.50 (10.14)	16.45 (10.70)	*F*_(2, 57)_ = 19.578; *p* < 0.0001	*p* < 0.0001	*p* < 0.0001	*p* = 0.520
IES–AVO	1.30 (1.59)	21.15 (9.25)	14.25 (10.69)	*F*_(2, 57)_ = 30.098; *p* < 0.0001	*p* < 0.0001	*p* < 0.0001	*p* = 0.031
BDI–TOT	2.10 (3.16)	19.40 (10.02)	13.45 (7.66)	*F*_(2, 57)_ = 27.422; *p* < 0.0001	*p* < 0.0001	*p* < 0.0001	*p* = 0.045
BDI–COG	1.05 (1.79)	12.50 (7.44)	8.95 (5.07)	*F*_(2, 57)_ = 24.462; *p* < 0.0001	*p* < 0.0001	*p* < 0.0001	*p* = 0.116
BDI–SOM	1.05 (1.67)	6.90 (3.26)	4.50 (2.97)	*F*_(2, 57)_ = 23.374; *p* < 0.0001	*p* < 0.0001	*p* < 0.0001	*p* = 0.022
SCL-90-R–GSI	0.94 (0.50)	1.31 (0.52)	1.08 (0.05)	*F*_(2, 57)_ = 3.888; *p* = 0.026	*p* = 0.023	*p* = 0.902	*p* = 0.274
SCL-90-R–PST	8.60 (8.83)	58.15 (20.30)	49.25 (25.44)	*F*_(2, 57)_ = 36.817; *p* < 0.0001	*p* < 0.0001	*p* < 0.0001	*p* = 0.461
SCL-90-R–PSDI	0.89 (0.49)	2.04 (0.51)	1.63 (0.42)	*F*_(2, 57)_ = 29.926; *p* < 0.0001	*p* < 0.0001	*p* < 0.0001	*p* = 0.024

Post-hoc test with Bonferroni adjustment for multiple comparisons is also reported. Data are mean (SD). ET, EMDR Treatment; ET × G, EMDR Treatment × Group. CAPS scores for RM group are available for only 10 subjects.

CAPS, Clinician-Administered PTSD Scale; CAPS–TOT, CAPS total score; CAPS–RE-EXP, CAPS re-experiencing symptoms; CAPS–AVO-NUM, CAPS avoidant-numbing symptoms; CAPS–H-AROU, CAPS hyper-arousal symptoms; CAPS–ASSOC, CAPS associated features; IES, Impact of Event Scale; IES–TOT, IES total score; IES–INT, IES intrusion symptoms; IES–AVO, IES avoidance symptoms; BDI, Beck Depression Inventory; BDI–TOT, BDI total score; BDI–COG, BDI cognitive symptoms; BDI–SOM, BDI somatic symptoms; SCL-90-R, Symptom CheckList-90-Revised; SCL-90-R–GSI, SCL-90-R Global Severity Index; SCL-90-R–PST, SCL-90-R Positive Symptom Total; SCL-90-R–PSDI, SCL-90-R Positive Symptom Distress Index.

As compared to T0, at T1 all scores decreased significantly in RM and so did, also if with some exception, in SGP (Table 3). None of the scores showed any significant group difference at T1 (Table 3).

**Table 3:** **Descriptive and rm-ANOVA (pre- vs. post-EMDR) statistics of psychopathological scales are showed**.

	**RM–pre (*n* = 20)**	**RM–post (*n* = 20)**	**SGP–pre (*n* = 20)**	**SGP–post (*n* = 20)**	**rm-ANOVAs**				
					**ET effect**	**ET × G effect**	**Bonferroni *post-hoc***		
							**RM–pre vs. RM–post**	**SGP–pre vs. SGP–post**	**RM–post vs. SGP–post**
CAPS–TOT	77.80 (17.77)	14.20 (9.73)	62.60 (23.99)	24.90 (17.92)	*F*_(1, 28)_ = 220.921; *p* < 0.0001	*F*_(1, 28)_ = 14.442; *p* = 0.0007	*p* < 0.0001	*p* < 0.0001	*p* = 0.951
CAPS–RE-EXP	17.90 (5.53)	3.00 (2.75)	13.25 (9.34)	3.55 (4.16)	*F*_(1, 28)_ = 97.626; *p* < 0.0001	*F*_(1, 28)_ = 4.362; *p* = 0.046	*p* < 0.0001	*p* < 0.0001	*p* = 1.000
CAPS–AVO-NUM	25.50 (7.32)	5.10 (4.86)	17.90 (8.49)	8.10 (6.65)	*F*_(1, 28)_ = 131.404; *p* < 0.0001	*F*_(1, 28)_ = 16.189; *p* < 0.0004	*p* < 0.0001	*p* < 0.0001	*p* = 1.000
CAPS–H-AROU	15.30 (4.50)	3.60 (2.32)	14.35 (5.63)	7.40 (4.80)	*F*_(1, 28)_ = 90.295; *p* < 0.0001	*F*_(1, 28)_ = 5.857; *p* = 0.022	*p* < 0.0001	*p* < 0.0001	*p* = 0.269
CAPS–ASSOC	7.30 (4.03)	0.70 (1.64)	8.00 (5.77)	0.40 (1.05)	*F*_(1, 28)_ = 50.106; *p* < 0.0001	*F*_(1, 28)_ = 0.249; *p* = 0.622	*p* = 0.002	*p* < 0.0001	*p* = 0.269
IES–TOT	41.65 (18.29)	11.40 (11.90)	30.70 (18.39)	18.45 (16.08)	*F*_(1, 38)_ = 72.929; *p* < 0.0001	*F*_(1, 38)_ = 13.082; *p* < 0.0009	*p* < 0.0001	*p* < 0.008	*p* = 1.000
IES–INT	20.50 (10.14)	5.45 (5.76)	16.45 (10.70)	8.55 (7.58)	*F*_(1, 38)_ = 73.133; *p* < 0.0001	*F*_(1, 38)_ = 7.098; *p* = 0.011	*p* < 0.0001	*p* = 0.001	*p* = 1.000
IES–AVO	21.15 (9.25)	6.00 (6.93)	14.25 (10.69)	9.90 (9.41)	*F*_(1, 38)_ = 44.831; *p* < 0.0001	*F*_(1, 38)_ = 13.752; *p* < 0.0007	*p* < 0.0001	*p* = 0.248	*p* = 1.000
BDI–TOT	19.40 (10.02)	9.95 (8.70)	13.45 (7.66)	7.40 (6.41)	*F*_(1, 38)_ = 36.963; *p* < 0.0001	*F*_(1, 38)_ = 1.779; *p* = 0.190	*p* < 0.0001	*p* = 0.011	*p* = 1.000
BDI–COG	12.50 (7.44)	6.30 (6.05)	8.95 (5.07)	4.30 (4.13)	*F*_(1, 38)_ = 31.201; *p* < 0.0001	*F*_(1, 38)_ = 0.637; *p* = 0.430	*p* < 0.0004	*p* < 0.01	*p* = 1.000
BDI–SOM	6.90 (3.26)	4.10 (3.26)	4.50 (2.97)	3.10 (2.49)	*F*_(1, 38)_ = 15.445; *p* < 0.0004	*F*_(1, 38)_ = 1.716; *p* = 0.198	*p* = 0.004	*p* = 0.430	*p* = 1.000
SCL-90-R–GSI	1.31 (0.52)	0.85 (0.43)	1.08 (0.05)	0.98 (0.34)	*F*_(1, 38)_ = 13.295; *p* < 0.0008	*F*_(1, 38)_ = 5.611; *p* = 0.023	*p* < 0.0008	*p* = 1.000	*p* = 1.000
SCL-90-R–PST	58.15 (20.30)	42.40 (20.51)	49.25 (25.44)	35.75 (25.30)	*F*_(1, 38)_ = 25.683; *p* < 0.0001	*F*_(1, 38)_ = 0.152; *p* = 0.699	*p* < 0.003	*p* = 0.012	*p* = 1.000
SCL-90-R–PSDI	2.04 (0.51)	1.55 (0.58)	1.63 (0.42)	1.26 (0.57)	*F*_(1, 38)_ = 22.969; *p* < 0.0001	*F*_(1, 38)_ = 0.503; *p* = 0.483	*p* = 0.002	*p* = 0.038	*p* = 0.524

Post-hoc test with Bonferroni adjustment for multiple comparisons is also reported. Data are mean (SD). ET, EMDR Treatment; ET × G, EMDR Treatment × Group. CAPS scores for RM group are available for only 10 subjects.

CAPS, Clinician-Administered PTSD Scale; CAPS–TOT, CAPS total score; CAPS–RE-EXP, CAPS re-experiencing symptoms; CAPS–AVO-NUM, CAPS avoidant-numbing symptoms; CAPS–H-AROU, CAPS hyper-arousal symptoms; CAPS–ASSOC, CAPS associated features; IES, Impact of Event Scale; IES–TOT, IES total score; IES–INT, IES intrusion symptoms; IES–AVO, IES avoidance symptoms; BDI, Beck Depression Inventory; BDI–TOT, BDI total score; BDI–COG, BDI cognitive symptoms; BDI–SOM, BDI somatic symptoms; SCL-90-R, Symptom CheckList-90-Revised; SCL-90-R–GSI, SCL-90-R Global Severity Index; SCL-90-R–PST, SCL-90-R Positive Symptom Total; SCL-90-R–PSDI, SCL-90-R Positive Symptom Distress Index.

### EEG (Table 4)

#### RM clients and SGP clients at T0 vs. controls

**Figure 1:**
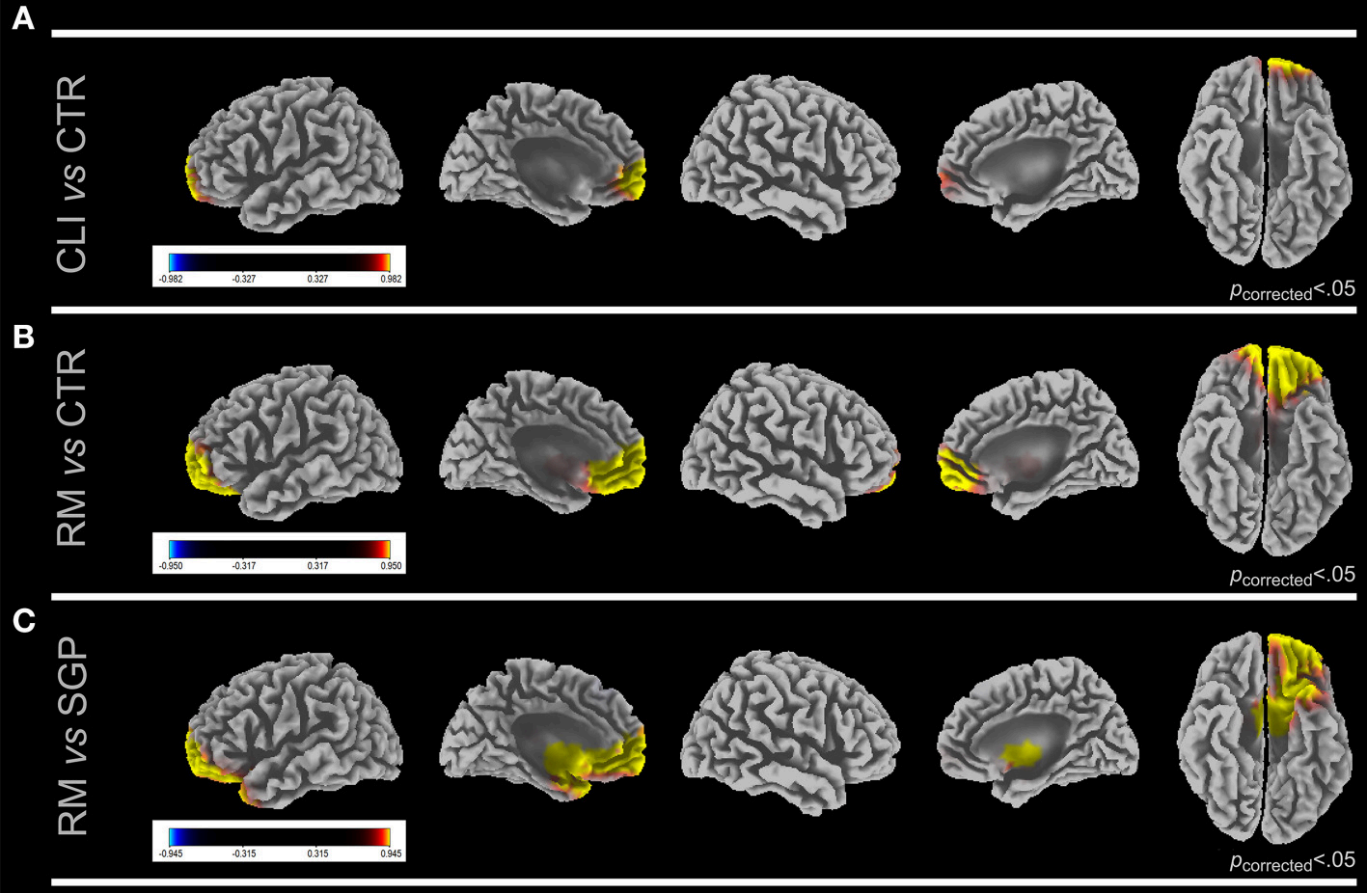
**LORETA images show cortical activation differences in gamma band at first session (T0) between CLI vs. CTR (A), RM vs. CTR (B), and RM vs. SGP, (C)**. Five views of the brain (lateral and medial left hemisphere, lateral and medial right hemisphere and ventral view) are shown for each comparison. Significant *F*-values (Bonferroni corrected) are reported. *F*-values colored scale is also shown for each comparison. Yellow, first group > second group; blue, second group > first group. CLI, All Clients; RM, Rome Clients; SGP, San Giuliano di Puglia Clients.

When all clients were compared to controls significantly higher cortical electrical activation was found upon BS in clients' left anterior frontal cortex (AFC; BAs 9, 10) and orbito-frontal cortex (OFC; BAs 11, 47) in γ band (Figure 1A). In the same condition all clients as compared to controls showed in α and β_1_ bands a lower activation than controls in right lateral temporal lobe (LTL; Bas 20, 21, 22, 38, 41, 42) and fusiform and lingual cortex (FLC; BAs 19, 37). In RM clients, activation in left AFC and OFC was significantly higher than in controls in γ band (Figure 1B). All other comparison did not result in any significant cortical electrical activation difference.

#### RM clients at T0 vs. SGP clients at T0

As compared to SGP clients RM clients showed a higher activation in left AFC and OFC in β_1_, β_2_, and γ bands (Figure 1C).

#### RM clients and SGP clients at T1 vs. controls

The only difference between clients and controls at T1 was the significantly higher activation in bilateral parahippocampal gyrus (PHG; BAs 27, 28, 34, 35, 36) in θ, α, and β_1_ bands in SGP clients as compared to controls.

#### RM clients at T1 vs. SGP clients at T1

As compared to SGP clients, RM clients showed at T1 a higher cortical electrical activity in PHG, LTL and posterior cingulate cortex (PCC; BAs 23, 29, 30, 31), particularly in θ and β_1_ bands. SGP clients significantly activated SFC and AFC more than RM clients in the γ band.

#### RM clients and SGP clients at T0 vs. T1

**Figure 2:**
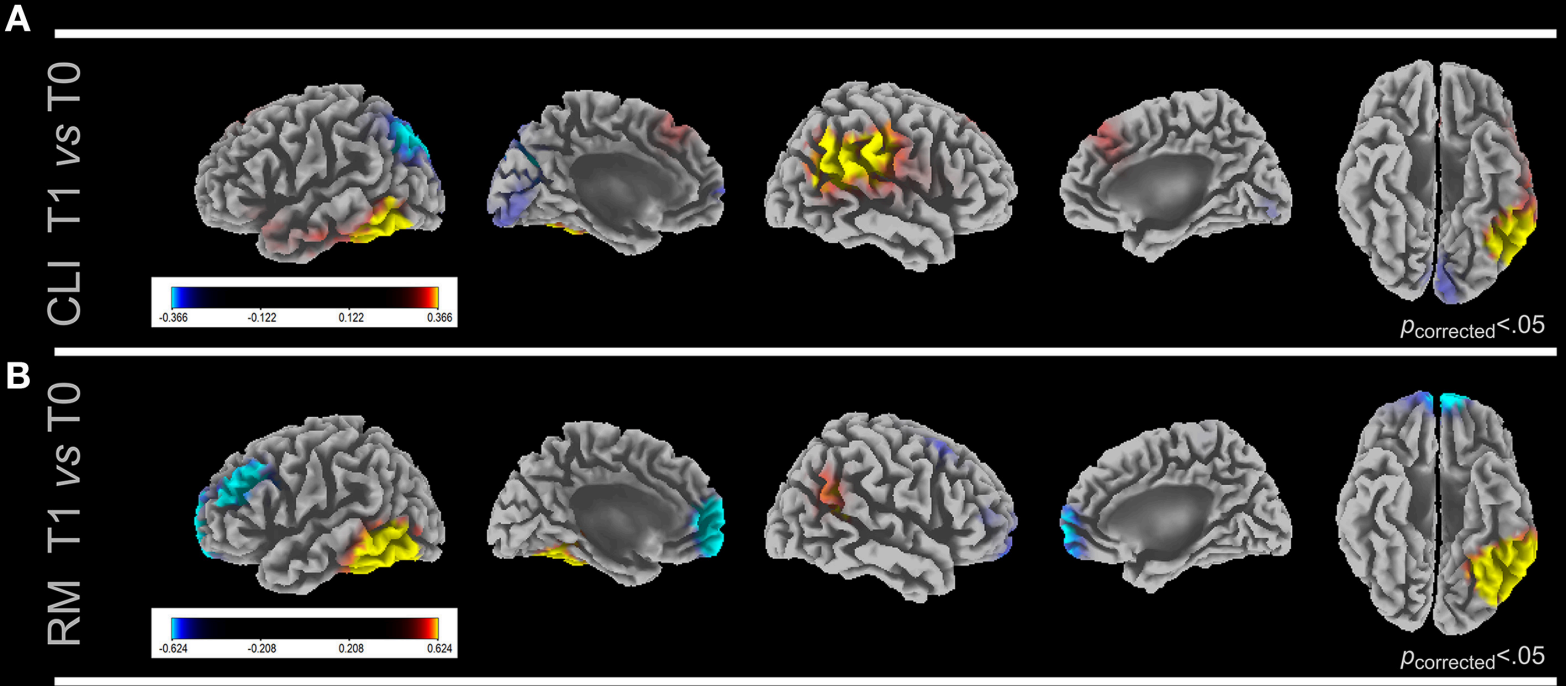
**LORETA images show cortical activation differences in gamma band between first (T0) and last (T1) sessions in CLI (A) and RM (B)**. Five views of the brain (lateral and medial left hemisphere, lateral and medial right hemisphere and ventral view) are shown for each comparison. Significant *F*-values (Bonferroni corrected) are reported. *F*-values colored scale is also shown for each comparison. yellow, first group > second group; blue, second group > first group. In **(A)** only the cluster depicted by yellow scale were found to be significant. CLI, All Clients; RM, Rome Clients; SGP, San Giuliano di Puglia Clients.

When all clients were compared between T0 and T1 a higher cortical signal was found at T0 in bilateral primary visual cortex (PVC; BAs 17, 18) in β_1_and β_2_ bands and in superior parietal lobule (SPL; BAs 5, 7) in β_2_ band (Figure 2A). An increased signal at T1 when compared to T0 was found in PHG in δ and θ band, and in left FLC and right inferior parietal lobule (IPL; BAs 39, 40, 43) in γ band in all clients. As for RM clients at T0 a significantly higher activation was found in left AFC in γ band and at T1 in FLC, both in δ and γ bands (Figure 2B). No differences were found in SGP clients in both comparisons.

## Discussion

To the best of our knowledge this is the first study to show distinct neurobiological responses during the reliving of major psychological trauma in two groups of psychologically traumatized clients, differing for trauma type, social and territorial context and exposure across time.

In the symptomatic phase all 40 clients grouped together showed a significantly increased activity upon the guided recall of their traumatic experiences during bilateral ocular stimulation as compared to controls in frontal and orbitofrontal cortex (Figure 1A; Table 4). During the same phase the detection, as compared to controls, in clients with mixed traumas (RM) of increased activity in γ band in the same regions (Figure 1B), and in clients exposed to natural disaster (SGP) of lack of cortical activation changes suggests the former group to be the major determinant of the differences found in all 40 clients grouped together. This is reinforced by the differences found in the direct comparison at T0 between SGP and RM showing a significantly higher activation in Anterior (Pre)Frontal Cortex and Orbital (Pre)Frontal Cortex mainly in γ band (Figure 1C). Also psychopathological evaluations confirmed at T0 this trend with lower scores for SGP as compared to the RM reaching in several comparisons significant difference (Table 2).

**Table 4:** **Regions of significant activation differences and relative cluster extent**.

	**ROI**	**DELTA**		**THETA**		**ALPHA**		**BETA1**		**BETA2**		**GAMMA**	
		***l***	***r***	***l***	***r***	***l***	***r***	***l***	***r***	***l***	***r***	***l***	***R***
T0 CLI vs. CTR	AFC											84	
	OFC											60	
T0 CTR vs. T0 CLI	OFC					56							
	LTL						81		55				
	FLC						61						
T0 RM vs. CTR	AFC											93	
	OFC											94	
T1 SGP vs. CTR	PHG			58		57	65	51	57				
T0 RM vs. T0 SGP	AFC							81		87		82	
	OFC							75		131		97	
T1 RM vs. T1 SGP	PHG	63	55	81	82		60	75	66				
	LTL			77				101					
	PCC			58	112			53	89				
T1 SGP vs. T1 RM	SFC											115	142
	AFC											59	119
T1 CLI vs. T0 CLI	PHG	63	73		74								
	FLC											68	
	IPL												69
T0 CLI vs. T1 CLI	PVC							84		144	102		
	SPL									97	78		
T1 RM vs. T0 RM	FLC	107	100									116	
T0 RM vs. T1 RM	AFC											69	

Values indicate the number of voxels with a p < 0.01 and a F-value over two z-score in clusters containing more than 50 voxels for a single region of interest. Regions of interest (ROI) with statistically significant differences pre-treatment (T0) and post-treatment (T1) between the whole group of 40 clients (CLI), the 20 Rome clients (RM), the 20 San Giuliano di Puglia clients (SGP), and the 20 controls (CTR). AFC, Anterior (Pre)Frontal Cortex; FLC, Fusiform and Lingual Cortex; IPL, Inferior Parietal Lobule; LTL, Lateral Temporal Lobe; OFC, Orbital (Pre)Frontal Cortex; PCC, Posterior Cingulate Cortex; PHG, ParaHippocampal Gyrus; PVC, Primary Visual Cortex; SFC, Superior (Pre)Frontal Cortex; SPL, Superior Parietal Lobule.

In PTSD prefrontal cortex hyperactivation was found during trauma recall (Shin et al., 1999; Lanius et al., 2002), has been associated to negative emotional states (Berkowitz et al., 2007), to amygdala hyperactivation (Gilboa et al., 2004) and to the inhibition of negative effects (Phan et al., 2005), correlated positively to heart rate changes (Barkay et al., 2012) and is involved in retrieval of emotional memories and extinction of conditioned fear (Milad and Rauch, 2007). The firing of prefrontal cortex upon traumatic memories represents the neurobiological correlate of the affective valence of the incoming information (Steele and Lawrie, 2004) and probably occurs when trauma processing has not taken place yet. Moreover, prefrontal cortex is implicated in autobiographical (Staniloiu et al., 2010) and episodic memory retrieval (Tulving et al., 1994) and suppresses unwanted memories (Anderson et al., 2004). In symptomatic clients these self-referential processes with high emotional contents may be overdone causing activation in prefrontal cortex larger than in normal individuals. In this respect EMDR has been proven to be effective in traumatized bipolar patients in whom it successfully modulated the changes occurring in Default Mode Network (Landin-Romero et al., 2013).

Consistently with previous studies, the relative psychotherapy-related activation decrease found in prefrontal cortex likely represents the successful down-regulation of emotional experiences. Possibly the forebrain cortical changes occurred during bilateral eye stimulation in the effort to encode unprocessed emotional material and it took place mainly in the γ band, known to be related in prefrontal cortex to the exposure to emotional stimuli (Hirata et al., 2007), speaking in favor of its modulation upon emotional processing. Neuronal firing in γ band in frontal cortex was also associated with behaviorally relevant sensory information and highly alert brain states (Fries et al., 2001) and was found to increase in response to sad stimuli (Ehlers et al., 2006).

At T1, in all 40 clients grouped together relative neuronal deactivation in primary visual cortex and superior parietal lobe and significantly higher cortical activation in fusiform and lingual cortex were detected as compared to T0 (Figure 2A). This trend was consistent with the pre- and post-treatment comparison in RM clients in which cortical activation shifted from Anterior (Pre)Frontal Cortex at T0 to fusiform and lingual cortex at T1 (Figure 2B). Moreover, post-treatment the differences in activation between RM clients and controls found at T0 disappeared. All these findings speak in favor of an EMDR-related response to reliving the traumatic event normalizing toward the one found in controls at T0, considered to be the gold standard for the neurophysiological response to the exposure to past psychological traumas. We can speculate that during EMDR memory retention of the traumatic event moves from regions with implicit emotional valence to association areas in which the experience is integrated and consolidated. In this respect the deactivation post-treatment (Figure 2A) of the primary visual cortex along with the activation of fusiform cortex implicated in the explicit representation of faces, words and abstract thoughts (Phillips et al., 2009) might be associated with an elaboration at higher cognitive level of the images related to the event.

At T1, when RM and SGP clients were compared a higher activity in the Anterior (Pre)Frontal Cortex and Superior (Pre)Frontal Cortex as well as a lower activity in ParaHippocampal Gyrus and Posterior Cingulate Cortex was found in the latter. This suggest that EMDR therapy resulted in the SGP clients, as compared to RM ones, in a attenuated disappearance of pre-treatment frontal activation and in a limited post-treatment cognitivization of the traumatic event. The post-treatment increase in ParaHippocampal Gyrus activation, mainly in theta band, in all 40 clients grouped together as compared to T0 and to controls might be related to the normalization in retrieving their own past, especially episodes of crucial importance for their affective-sensitive mnestic processing (Fink et al., 1996). Theta rhythm is implicated in episodic memory (Hasselmo, 2005) and memory formation and retrieval (Rutishauser et al., 2010) and has been found in hippocampus to increase in PTSD (Begic et al., 2001).

Taken together the results indicate that, despite the severe psychological trauma suffered by single individuals, SGP clients showed a lower functional response to traumatic exposure during EMDR therapy. Although symptom disappearance in SGP clients was associated to normalization of neuropsychiatric scores, functional activation in response to trauma reliving was not statistically different from that found in the symptomatic phase, also reflecting a milder neurobiological impact of the index trauma. Having occurred in a small town the event did involve the whole community and was a recurrent topic openly discussed for the last 10 years. Furthermore, images related to the event could not be avoided being part of the external environment. A possible explanation for the lack of significant neurobiological correlates of such strong emotional event lies in the fact that the recruited SGP clients as compared to the RM ones have been chronically exposed to the grief over time. Sharing the traumatic memory with other members of the family and of the community also led to experiencing components of social defeat and interpersonal distress (Krabbendam et al., 2014). This implied an ongoing trauma processing which made the clients cope daily with the losses and grief, and partially extinguish fear-inducing situations resulting in more structured traumatic memories with strong social implications and difficult to be accessed by psychotherapy, although EMDR could relieve all clients from symptoms. As a consequence, the activation of the prefrontal emotional cortex during the symptomatic phase was significantly lower in SGP clients than in RM ones and did not significantly differ from that of normal controls exposed to a traumatic event in which rationality overcame vivid emotions.

In this respect a recent investigation has shown upon retrieval of negative memories a lower amygdala activation in older adults as compared to younger ones. This would speak in favor of a time-related adaptation to emotional events occurring during life in which longer exposure to emotional events might result in a successful regulation of affective responses to negative stimuli (Ge et al., 2014).

During the symptomatic phase the cortical activation in all 40 clients grouped together in Lateral Temporal Lobe and Fusiform and Lingual Cortex was significantly lower than in controls (Table 4), suggesting that in the latter group exposure to traumatic events resulted in a prevalent activation of brain cortex with cognitive relevance instead of brain areas associated with emotions and behavior. Consistently, in response to negative emotions Cohen et al. (2013) found in control subjects an activation of inferior and middle temporal gyri and in PTSD patients a maximal activation in frontal cortex. The disappearance in all clients at T1 of the cortical hyperactivation in prefrontal regions present at T0 is the most relevant functional correlate of EMDR, attenuating the negative emotional response to the index trauma and impacting on the neurobiology of brain regions implicated in fear processing. Frontal cortex deactivation after successful psychotherapy is consistent with recent findings of efficacy of cognitive behavioral therapy in reducing the activation of the neural network of fear conditioning in panic disorder (Kircher et al., 2013). Forebrain is a key substrate of conscious experience with emotional valence monitoring information with affective consequences (Dalgleish, 2004) and is also associated with the processing of self-generated thoughts (Ramnani and Owen, 2004). The differential neuronal firing at T0 in all 40 clients grouped together as compared to control subjects in frontal and prefrontal cortex not only highlighted the emotional component of the trauma reliving when clients were still symptomatic but also ruled out the possibility that these regions were activated merely due to the autobiographic retrieval of the index event and/or due to the bilateral ocular stimulation. The greater difference between clients and controls subjects was not due to the nature of traumas but to the lack of symptoms in the latter. Physical and/or psychological traumas cause PTSD or anxiety states based not only on their severity but also on personality, on life-time trauma load and probably on genetic factors associated to each individual.

The shift of the prevalent cortical firing during trauma reliving from prefrontal regions with limbic valence at T0 to regions with more cognitive valence at T1 is the second relevant neurobiological correlate of processing associated with EMDR, and it is clinically confirmed by the highly significant decrease of all neuropsychological scores in RM clients and of the great majority of them in SGP clients.

The results confirmed the findings of a preliminary investigation reporting in real-time the cortical activation changes occurring during EMDR therapy (Pagani et al., 2012). In the present study performed in a three times larger cohort of subjects the previous findings were indeed strengthened decreasing the likelihood of type II error since the sampling error decreases with the increase of the sample size. The EEG recordings were carried out in client-friendly environments, avoiding possible biases due to physical and psychological discomfort to the clients caused by a non-natural noisy examination condition, resulting in unspecific activations (Mazard et al., 2002). The dynamic representation of psychotherapy on the cerebral cortex by functional imaging is by now an *unicum* in neuroimaging and paves the road to investigations implying longitudinal monitoring and therapeutic interventions during sessions.

We also acknowledge some limitations of the study. Firstly, the spatial resolution of a 37-channel EEG device is too low to allow a reliable detection of changes occurring in amygdala and central structures, limiting the analyses to the cortical regions. Secondly, the possibility to subgroup SGP clients is prevented by a scarce numerosity not allowing further reliable statistical analyses and inferences about the neurobiological differences between clients having been exposed to the traumatic event in the same social context and for the same number of years but with different types of trauma (direct vs. indirect). Thirdly, 10 clients did not satisfy the full criteria for current PTSD as evaluated by MINI-Plus (Table 1). However, all of them were exposed to a major traumatic event, were symptomatic and the scores of most of the psychopathological evaluations and clinical examination classified them as suffering moderate to extreme PTSD (see Table 2). This sample can then be considered representative of severely psychological traumatized individuals not differing from the majority of subjects recruited in the past investigations on PTSD.

EEG monitoring enabled for the first time to disclose the neurobiological differences between groups of clients traumatized in different social and territorial context during the reliving of the traumatic event. The group chronically exposed to traumatic memories following a natural disaster showed moderate response to psychopathological evaluations and event reliving, speaking in favor of an impact on trauma neurobiology of social and environmental contexts.

### Conflict of interest statement

The Review Editor France Haour declares that, despite being affiliated to the same association as author Isabel Fernandez, the review process was handled objectively and no conflict of interest exists. Isabel Fernandez is the President of the Italian EMDR Association and EMDR Europe Association. The authors declare that the research was conducted in the absence of any commercial or financial relationships that could be construed as a potential conflict of interest.
